# A Robust Shape Reconstruction Method for Facial Feature Point Detection

**DOI:** 10.1155/2017/4579398

**Published:** 2017-02-19

**Authors:** Shuqiu Tan, Dongyi Chen, Chenggang Guo, Zhiqi Huang

**Affiliations:** School of Automation Engineering, University of Electronic Science and Technology of China, No. 2006, Xiyuan Ave, West Hi-Tech Zone, Chengdu 611731, China

## Abstract

Facial feature point detection has been receiving great research advances in recent years. Numerous methods have been developed and applied in practical face analysis systems. However, it is still a quite challenging task because of the large variability in expression and gestures and the existence of occlusions in real-world photo shoot. In this paper, we present a robust sparse reconstruction method for the face alignment problems. Instead of a direct regression between the feature space and the shape space, the concept of shape increment reconstruction is introduced. Moreover, a set of coupled overcomplete dictionaries termed the shape increment dictionary and the local appearance dictionary are learned in a regressive manner to select robust features and fit shape increments. Additionally, to make the learned model more generalized, we select the best matched parameter set through extensive validation tests. Experimental results on three public datasets demonstrate that the proposed method achieves a better robustness over the state-of-the-art methods.

## 1. Introduction

**Figure 1:**
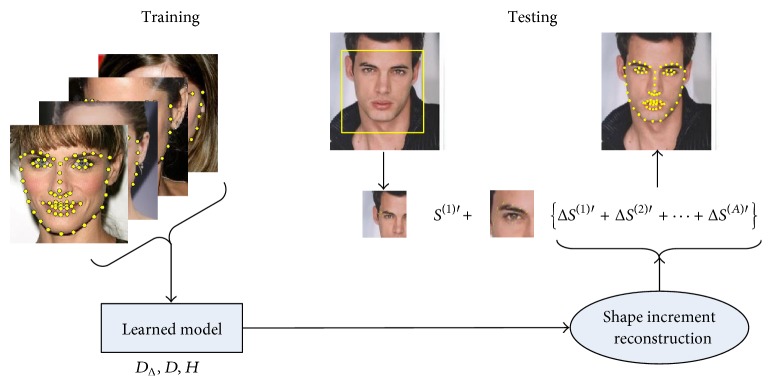
Schematic diagram of our robust sparse reconstruction method for facial feature point detection.

In most literatures, facial feature points are also referred to facial landmarks or facial fiducial points. These points mainly locate around edges or corners of facial components such as eyebrows, eyes, mouth, nose, and jaw (see Figure 1). Existing databases for method comparison are labeled with different number of feature points, varying from the minimum 5-point configuration [1] to the maximal 194-point configuration [2]. Generally facial feature point detection is a supervised or semisupervised learning process that trains model on a large number of labeled facial images. It starts from a face detection process and then predicts facial landmarks inside the detected face bounding box. The localized facial feature points can be utilized for various face analysis tasks, for example, face recognition [3], facial animation [4], facial expression detection [5], and head pose tracking [6].

In recent years, regression-based methods have gained increasing attention for robust facial feature point detection. Among these methods, a cascade framework is adopted to recursively estimate the face shape *S* of an input image, which is the concatenation of facial feature point coordinates. Beginning with an initial shape *S*^(1)^, *S* is updated by inferring a shape increment Δ*S* from the previous shape:

(1) $$\begin{array}{c} \Delta S^{\left(t\right)}=W^{\left(t\right)}\Phi^{\left(t\right)}\left(\;I,S^{\left(t\right)}\;\right), \end{array}$$

where Δ*S*^(*t*)^ and *W*^(*t*)^ are the shape increment and linear regression matrix after *t* iterations, respectively. As the input variable of the mapping function Φ^(*t*)^, *I* denotes the image appearance and *S*^(*t*)^ denotes the corresponding face shape. The regression goes to the next iteration by the additive formula:

(2) $$\begin{array}{c} S^{\left(t\right)}=S^{\left(t-1\right)}+\Delta S^{\left(t-1\right)}. \end{array}$$

In this paper, we propose a sparse reconstruction method that embeds sparse coding in the reconstruction of shape increment. As a very popular signal coding algorithm, sparse coding has been recently successfully applied to the fields of computer vision and machine learning, such as feature selection and clustering analysis, image classification, and face recognition [7–11]. In our method, sparse overcomplete dictionaries are learned to encode various facial poses and local textures considering the complex nature of imaging conditions. The schematic diagram of the proposed shape increment reconstruction method is illustrated in Figure 1. In the training stage, two kinds of overcomplete dictionaries need to be learned. The first kind of dictionary is termed shape increment dictionary since the atoms consist of typical shape increments in each iteration. The other kind of dictionary is termed local appearance dictionary because of the atoms abstracting the complex facial feature appearance. In the testing stage, local features are extracted around the shape points of current iteration and then encoded into feature coefficients using the local appearance dictionary. Thus shape increments can be reconstructed by the shape increment dictionary and the shape coefficients transformed from the feature coefficients. Considering the holistic performance, we adopt a way of alternate verification and local enumeration to get the best parameter set in a large number of experiments. Comparison with three previous methods is evaluated on three publicly available face datasets. Experimental results show that the proposed sparse reconstruction method achieves a superior detection robustness comparing with other methods.

The following contents of this paper are organized as follows: related work is introduced in Section 2. The proposed sparse reconstruction method is described in detail in Section 3 and experimental results are compared in Section 4. Finally we conclude the whole paper in Section 5.

## 2. Related Work

During the past two decades, a large number of methods have been proposed for facial feature point detection. Among the early methods, Active Appearance Model (AAM) [12] is a representative parametric model that aims to minimize the difference between the texture sampled from the testing image and the texture synthesized by the model. Later many improvements and extensions of AAM are proposed [6, 13–20]. To improve the efficiency in real-time system, Tzimiropoulos and Pantic [16] proposed a model to efficiently solve the AAM fitting problem. Tresadern et al. [14] used Haar-like features to reduce computation, which can help the mobile device to perform real-time tracking. Nguyen et al. [17–19] thought AAMs are easily converged to local minima. And to overcome this problem, they designed a new model that learns a cost function having local minima only at desired places. In terms of improving robustness, Huang et al. [6] combined view-based AAM [20] and Kalman filter to perform pose tracking and use shape parameters to rebuild the view space. Hansen et al. [13] introduced a nonlinear shape model that based on Riemannian elasticity model to handle the problem of poor pose initialization.

Generally, Constrained Local Model- (CLM-) based methods [21–25] are to learn a group of local experts and then take various shape prior for refinement. Vogler et al. [21] used Active Shape Model (ASM) [23] to build a 3D deformable model for real-time tracking. Yu et al. [22] used the mean-shift method [25] to rapidly approach the global optimum. And Liang et al. [24] constrained the structure of facial feature points using the component locations.

The aforementioned methods share the same characteristic which controls face shape variations through some certain parameters. But different from those methods, the regression-based methods [26–28] directly learn the regression function from image appearance to target output. Gao et al. [26] adopt a two-level cascaded boosted regression [27] structure to obtain a vectorial output for all points. To solve the problems of large shape variations and occlusions, Burgos-Artizzu et al. [28] improved their method in three aspects: firstly, they first reference pixels by linear interpolation between two landmarks. Secondly, the regression model directly embeds the occlusion information for robustness. Thirdly, they designed a smart initialization restart scheme to avoid unsuitable random initializations.

Our method belongs to the regression-based method, like [28–31]. However, our work is different from previous methods in several aspects. Firstly, existing methods, like [31], acquire the descent direction in a supervised learning manner. But in our proposed method, the information of descent direction is included in the sparse dictionaries for reconstructing the shape increment. And then unlike the method in [28], our method has no usage of the occlusion information of each feature point. Finally the method from [30] designed a two-level boosted regression model to infer the holistic face shape. In our regression model, we refine the face shape by the learned two coupled dictionaries stage by stage.

## 3. Regression-Based Sparse Reconstruction Method

### 3.1. Problem Formulation

In this paper, the alignment target of all methods is assessed through the following formula:

(3) $$\begin{array}{c} {\left\|\;S^{\left(final\right)}-S^{\left(ground\text{-}truth\right)}\;\right\|}_{2}, \end{array}$$

where *S*^(final)^ and *S*^(ground-truth)^ denote the final estimated shape and the corresponding ground-truth shape of an image, respectively. In the regression-based methods, the iterative equation is formulated as the following:

(4) $$\begin{array}{c} S^{\left(k\right)}=S^{\left(k-1\right)}+\Delta S^{\left(k-1\right)*}; \end{array}$$

here Δ*S*^(*k*−1)*∗*^ is a variable of shape increment after *k* − 1 iterations, and its value should approximate the ground-truth shape increment Δ*S*^(*k*−1)^, where Δ*S*^(*k*−1)^ = *S*^(ground-truth)^ − *S*^(*k*−1)^.

### 3.2. Multi-Initialization and Multiparameter Strategies

Multi-initialization means diversification of initial iteration shape which can improve robustness of the reconstruction model. Specifically, we randomly select multiple ground-truth face shapes from the training set to form a group of initial shapes for the current image. Obviously, the multi-initialization strategy is able to enlarge the training sample size and enrich extracted feature information that makes each regression model more robust, while, during the testing stage, multi-initialization can create more chances to step out of potential local minima that may lead to inaccurate feature point localization.

In our method, there are four key parameters that are the size of feature dictionary, the size of shape increment dictionary, and their corresponding sparsity. The selection of the four parameters has a direct influence on the learned reconstruction model. Therefore we do a large number of validation tests to find the best matched parameters. Then according to the validation results, we decide to adopt three sets of parameters to train the model.

### 3.3. The Learning of Sparse Coding

We use the Orthogonal Matching Pursuit (OMP) [32] algorithm and the* K*-Singular Value Decomposition (*K*-SVD) [33] algorithm to find the overcomplete dictionary by minimizing the overall reconstruction error:

(5) minD,γ S−DγF2subject  to γi0≤T0,∀i,

where *S* is the input data and *D* and *γ* denote sparse dictionary and sparse coefficient, respectively. *T*_0_ defines the number of nonzero values in a coefficient vector and is termed the sparsity.

### 3.4. The Learning of Shape Increment

In Supervised Descend Method (SDM [31]), authors adopt a linear regression equation to approximate shape increments:

(6) $$\begin{array}{c} \Delta S^{\left(k\right)}=R^{\left(k\right)}\phi^{\left(k\right)}+b^{\left(k\right)}; \end{array}$$

here *ϕ*^(*k*)^ denotes Histograms of Oriented Gradients (HoG) features extracted from the shapes *S*^(*k*)^ of previous stage. *R*^(*k*)^ and *b*^(*k*)^ are got from the training set by minimizing

(7) $$\begin{array}{c} \underset{R^{\left(k\right)},b^{\left(k\right)}}{\arg\;\min}\underset{i}{\sum }{\left\|\;\Delta S^{\left(ki\right)*}-R^{\left(k\right)}\phi^{\left(ki\right)}-b^{\left(k\right)}\;\right\|}_{2}^{2}. \end{array}$$

Different from the idea of linear approximation proposed in SDM, we introduce the concept of direct sparse reconstruction for reconstructing shape increments:

(8) $$\begin{array}{c} \Delta S^{\left(k\right)*}=D_{\Delta }^{\left(k\right)}\gamma_{\Delta }^{\left(k\right)}. \end{array}$$

Here *D*_Δ_^(*k*)^ and *γ*_Δ_^(*k*)^ represent the shape increment dictionary and its corresponding sparse coefficient in the *k*th iteration, respectively. From another perspective the generic descent directions are embedded into the sparse dictionary *D*_Δ_^(*k*)^ which can be more robust in facing large shape variations.

### 3.5. The Shape Regression Framework

To better represent local appearances around facial feature points, the extracted HoG features are also encoded into sparse coefficients:

(9) $$\begin{array}{c} \phi^{\left(k\right)}=D^{\left(k\right)}\gamma^{\left(k\right)}, \end{array}$$

where *D*^(*k*)^ and *γ*^(*k*)^ are the local appearance dictionary and the local appearance sparse coefficient, respectively. Instead of a direct mapping from the whole feature space to the shape increment space, we propose to perform regression only in the sparse coefficient space. Since both coefficient matrixes are sufficient sparse, the regression matrix can be quickly solved. The equation is formulated as follows:

(10) $$\begin{array}{c} \gamma_{\Delta }^{\left(k\right)}=H^{\left(k\right)}\gamma^{\left(k\right)}. \end{array}$$

Now we describe the shape regression framework in detail (see Pseudocode 1). During the training stage, we can get the shape prediction of the next stage using (4). By iterative learning shape increment Δ*S*^(*k*)*∗*^, we can obtain the final face shape. Combining (10) and (8) Δ*S*^(*k*)*∗*^ is computed from Δ*S*^(*k*)*∗*^ = *D*_Δ_^(*k*)^*H*^(*k*)^*γ*^(*k*)^, where *D*_Δ_^(*k*)^ and *γ*^(*k*)^ are variables that can be acquired by the following sparse reconstruction formulas:

(11) arg minDk,γk ϕk−Dkγk22,s.t γk0≤Tϕarg minDΔk,γΔk ΔSk−DΔkγΔk22,s.t γΔk0≤TΔ.

*T*_Δ_ and *T*_*ϕ*_ represent the shape increment sparsity and the local appearance sparsity, respectively. Given *γ*^(*k*)^ and *γ*_Δ_^(*k*)^ we can get *H*^(*k*)^ by

(12) $$\begin{array}{c} \underset{H^{\left(k\right)}}{\arg\;\min}{\left\|\;\gamma_{\Delta }^{\left(k\right)}-H^{\left(k\right)}\gamma^{\left(k\right)}\;\right\|}_{2}^{2}. \end{array}$$

Finally, we can generate a set of [*D*_Δ_^(1)^, *D*_Δ_^(2)^,…, *D*_Δ_^(*Q*)^], [*D*^(1)^, *D*^(2)^,…, *D*^(*Q*)^] and [*H*^(1)^, *H*^(2)^,…, *H*^(*Q*)^] after *Q* iterations. Here *Q* is the number of iterations and *k* = 1,2,…, *Q*.

During the testing stage, we can get the local appearance coefficients *γ*^(*k*−1)′^ using the already learned *D*^(*k*−1)^. Then the final face shape is estimated using (16) and (17) after *Q* iterations.

(13) Sk+1′=Sk′+ΔSk′,

(14) ΔSk+1′=DΔkHkγk′.

### 3.6. Major Contributions of the Proposed Method

In this section, we summarize the following three contributions of the proposed method:

1. Sparse coding is utilized to learn a set of coupled dictionaries, named the shape increment dictionary and the local appearance dictionary. The solved corresponding sparse coefficients are embedded in a regression framework for approximating the ground-truth shape increments.
2. A way of alternate verification and local enumeration is applied for selecting the best parameter set in extensive experiments. Moreover, it is shown in experimental results that the proposed method has a strong stability under different parameter settings.
3. We also rebuild testing conditions that the top 5%, 10%, 15%, 20%, and 25% of the testing images are removed according to the descending order sorted by the normalized alignment error. And then the proposed method is compared with three classical methods on three publicly available face datasets. Results support that the proposed method achieves better detection accuracy and robustness than the other three methods.

## 4. Experiments

### 4.1. Face Datasets

In this section, three publicly available face datasets are selected for performance comparison: Labeled Face Parts in the Wild (LFPW-68 points and LFPW-29 points [16]) and Caltech Occluded Faces in the Wild 2013 (COFW) [34]. In the downloaded LFPW dataset, 811 training images and 224 testing images are collected. Both the 68 points' configuration and the 29 points' configuration labeled for the LFPW dataset are evaluated. The COFW dataset includes 1,345 training images and 507 testing images, and each image is labeled with 29 facial feature points and related binary occlusion information. Particularly, collected images in this dataset show a variety of occlusions and large shape variations.

### 4.2. Implementation Details

#### 4.2.1. Codes

The implementation codes of SDM [31], Explicit Shape Regression (ESR) [30], and Robust Cascaded Pose Regression (RCPR) [28] are got from the Internet. Except that the codes of RCPR and ESR are released on the personal websites by at least one of the authors, we get the code of SDM from Github.

#### 4.2.2. Parameter Settings

Generally, the size of shape increment dictionary and local appearance dictionary in our method depends on the dimensionality of the HoG descriptor. And in the following validation experiments, we will introduce how to select the best combination of parameters. Parameters settings of SDM, ESR, and RCPR are consistent with the original settings reported in the papers. In SDM, the regression runs 5 stages. In ESR, the number of features in a fern and candidate pixel features are 5 and 400, respectively. To build the model, the method uses 10 and 500 stages to train a two-level boosted framework. And in RCPR, 15 iterations, 5 restarts, 400 features, and 100 random fern regressors are adopted.

#### 4.2.3. Assessment Criteria

In our experiments, we use the following equation to calculate and normalize the alignment errors. Firstly, we calculate the localization errors between the ground-truth point coordinates and the detected point coordinates, that is, the Euclidean distance between two vectors. Then it is further normalized by the interocular distance as follows:

(15) $$\begin{array}{c} d_{error}=\frac{{\left\|P-G\right\|}_{2}}{{\left\|G_{leye}-G_{reye}\right\|}_{2}}. \end{array}$$

In (15), *P* denotes the detected facial point coordinates and *G* denotes the ground-truth point coordinates. *G*_leye_ and *G*_reye_ denote the ground-truth center coordinates of left eye and right eye, respectively.

### 4.3. Experiments

#### 4.3.1. Parameter Validation

In this section, we will introduce how to use the way of alternate verification and local enumeration to find the final values of parameters. As described above, there are six variables *T*_Δ_, *T*_*ϕ*_, size of *D*_Δ_, size of *D*, *Q*, and *K* that need to be fixed; here *K* is the initialization number during the process of training and testing. Depending on the requirements of sparsity, the candidate values of *T*_Δ_ and *T*_*ϕ*_ are selected from the following set:

(16) $$\begin{array}{c} T_{\Delta },T_{\phi }\in \left\{\;2,4,6,8,10\;\right\}. \end{array}$$

Similarly, the candidate sizes of *D*_Δ_, sizes of *D*, *Q*, and *K* form the following sets:

(17) size  of  DΔ∈256,512,size  of  D∈256,512,Q∈1,2,3,4,5,K∈1,6,8,10,12,14.

Firstly the values of *Q* and *K* are set to 5 and 1, respectively. Note that the value of *K* in the testing stage should be equal to the value of *K* in the training stage. Then we set the value of *T*_*ϕ*_ to 2, 4, 6, 8, and 10 sequentially. The size of *D*_Δ_ and *D* is selected in random combination. For different values of *T*_Δ_ we can get five groups of results. And Table 1 gives the detailed results when *T*_Δ_ is fixed to 2. From Table 1 we may find that the parameter set {2,8, 256,256,5, 1  &  1} achieves the lowest alignment error. Similarly we conduct the rest experiments and find the best parameter sets. The corresponding sparsity is also fixed and therefore we get three sets of parameters that are {6,10,512,256}, {8,8, 512,256}, and {10,10,512,256}. In Table 2, we test the multi-initialization and multiparameter strategies while the regression runs 4 iterations and 10 initializations with different parameter settings. In the final, all point localizations are averaged to get the fusion result.

#### 4.3.2. Comparison with Previous Methods

**Figure 2:**
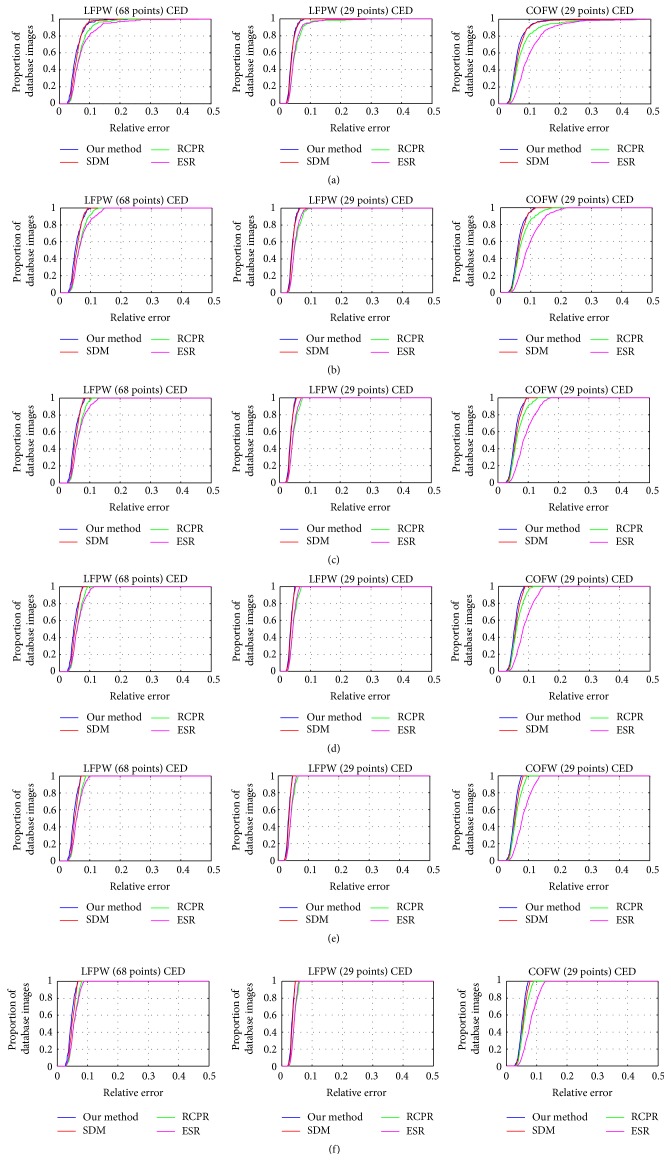
Cumulative Error Distribution (CED) curves of four methods tested on LFPW (68 points), LFPW (29 points), and COFW (29 points) datasets. The top (a) 0%, (b) 5%, (c) 10%, (d) 15%, (e) 20%, and (f) 25% of the testing images are removed according to the descending order sorted by the normalized alignment errors.

Due to the existence of a small number of facial images having large shape variations and severe occlusions, it challenges the random multi-initialization strategy which fails to generate an appropriate starting shape. Therefore we compare our method with three classic methods on rebuilt datasets. These datasets still include most of the images coming from LFPW (68 points), LFPW (29 points), and COFW (29 points). We just remove the top 5%, 10%, 15%, 20%, and 25% of the testing facial images in each dataset by sorting the alignment errors in a descending order (see Figure 2).

In Figure 2, all curves of COFW show a more dispersive distribution than the other two datasets. Since this dataset consists of many more facial images with large shape variations and occlusions, it may affect the detection accuracy more or less. Meanwhile, the irregular textural features around facial feature points are challenging for learning of structural model during the training. Obviously, in Figure 2, the curves of our proposed method are superior to the others. Additionally the LFPW (68 points) and LFPW (29 points) share the same facial images but different face shapes, so we may find some useful information about the performance of methods through these datasets.

In general, the more facial feature points are, the more difficult they are to detect. By comparing among five facial components, the mean errors of nose and eyes given in Tables 3 and 4 do not change obviously across three datasets, because the vicinal textural information of eyes is easy to recognize and the textural information around nose has a less possibility to be occluded. Moreover, the facial feature points located in the regions of nose and eyes are denser than the points of contour, which is also benefit to the regressive searching process.

**Figure 3:**
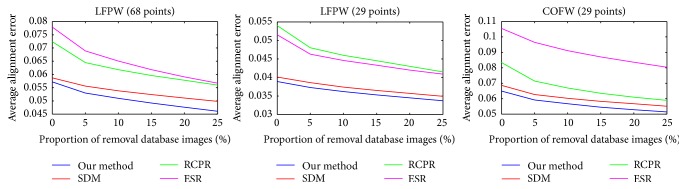
Mean errors of four methods tested on LFPW (68 points), LFPW (29 points), and COFW (29 points) datasets.

Figure 3 shows the alignment errors of four methods tested on LFPW (68 points), LFPW (29 points), and COFW (29 points) datasets. In Figure 3 we may find that the mean error curves show a rapid descending trend when the most difficult 5% of testing images are removed. It indicates that the statistical average can be biased by a few challenge images. Then as the removal proportion increases, all the curves become smoother. It shows in Figure 3 that our proposed method is more stable than other methods, which means our training model has a robustness in dealing with occlusions and large shape variations.

**Figure 4:**
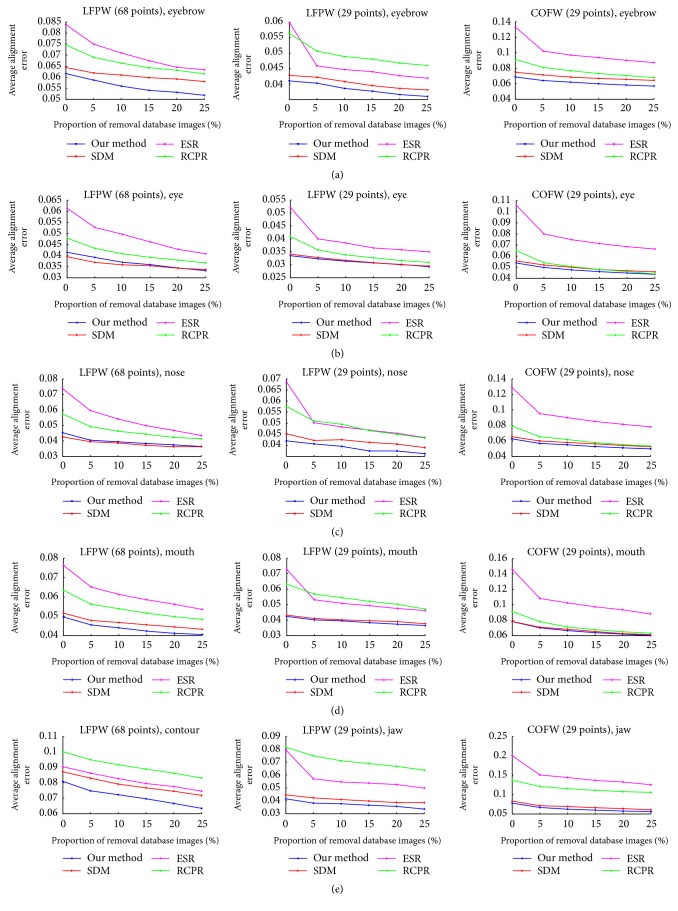
Facial feature point detection curves of four methods for each facial component on LFPW (68 points), LFPW (29 points), and COFW (29 points) datasets. (a) Eyebrow. (b) Eye. (c) Nose. (d) Mouth. (e) Contour or jaw.

Specifically, we plot the detection curves of five facial components in Figure 4. It is obvious in Figure 4 that ESR and RCPR has a less competitive performance for localizing each facial components. And our method shows better robustness in localizing feature points that belong to eyebrows and contour, since these two facial components are very likely to be occluded by hair or objects and have a more separated distribution pattern. Experimental results demonstrates that our proposed method can estimate facial feature points with high accuracy and is able to deal with the task of face alignment on complex occlusions and large shape variations.

## 5. Conclusion

A robust sparse reconstruction method for facial feature point detection is proposed in this paper. In the method, we build the regressive training model by learning a coupled set of shape increment dictionaries and local appearance dictionaries which are learned to encode various facial poses and rich local textures. And then we apply the sparse model to infer the final face shape locations of an input image by continuous reconstruction of shape increments. Moreover, in order to find the best matched parameters, we perform extensive validation tests by using the way of alternate verification and local enumeration. It shows in the comparison results that our sparse coding based reconstruction model has a strong stability. In the later experiments, we compare our proposed method with three classic methods on three publicly available face datasets when removing the top 0%, 5%, 10%, 15%, 20%, and 25% of the testing facial images according to the descending order of alignment errors. The experimental results also support that our method is superior to the others in detection accuracy and robustness.

## Figures and Tables

**Pseudocode 1:**
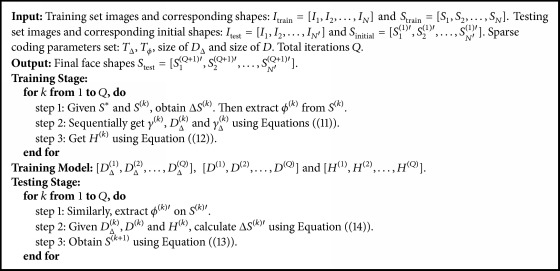
Pseudocode of our proposed regression-based method.

**Table 1:** Comparison of different parameter sets on LFPW (68 points) dataset. Here *T*_Δ_ is fixed to 2.

*T*_Δ_	*T*_*ϕ*_	Size of *D*_Δ_	Size of *D*	*Q*	*K* (tr & ts)	Mean errors
2	2	256	256	5	1 & 1	0.079381
		256	512	5	1 & 1	0.08572
		512	256	5	1 & 1	0.085932
		512	512	5	1 & 1	0.086731

2	4	256	256	5	1 & 1	0.081958
		256	512	5	1 & 1	0.083715
		512	256	5	1 & 1	0.086187
		512	512	5	1 & 1	0.087157

2	6	256	256	5	1 & 1	0.07937
		256	512	5	1 & 1	0.07986
		512	256	5	1 & 1	0.075987
		512	512	5	1 & 1	0.084429

2	8	256	256	5	1 & 1	0.075863
		256	512	5	1 & 1	0.082588
		512	256	5	1 & 1	0.077048
		512	512	5	1 & 1	0.082644

2	10	256	256	5	1 & 1	0.076178
		256	512	5	1 & 1	0.076865
		512	256	5	1 & 1	0.080907
		512	512	5	1 & 1	0.088414

**Table 2:** Comparison of multi-initialization and multiparameter strategies on LFPW (68 points) dataset. Here *Q* and *K* are set to 4 and 10, respectively.

*T*_Δ_	*T*_*ϕ*_	Size of *D*_Δ_	Size of *D*	*Q*	*K* (tr & ts)	Mean errors	Fusion errors
6	10	512	256	4	10 & 10	0.062189	0.055179
10	10	512	256	4	10 & 10	0.06075	
8	8	512	256	4	10 & 10	0.061787	

**(a):** 

	Method	Contour	Eyebrow	Mouth	Nose	Eye
LFPW (68 points)	SDM	0.0829	0.0619	0.0478	0.0395	0.0369
	ESR	0.0862	0.0750	0.0651	0.0596	0.0527
	RCPR	0.0948	0.0690	0.0562	0.0493	0.0433
	Our method	0.0747	0.0587	0.0455	0.0405	0.0392

**(b):** 

	Method	Jaw	Eyebrow	Mouth	Nose	Eye
LFPW (29 points)	SDM	0.0422	0.0422	0.0410	0.0422	0.0328
	ESR	0.0570	0.0459	0.0531	0.0502	0.0400
	RCPR	0.0748	0.0507	0.0568	0.0509	0.0357
	Our method	0.0382	0.0403	0.0401	0.0406	0.0323

**(c):** 

	Method	Jaw	Eyebrow	Mouth	Nose	Eye
COFW (29 points)	SDM	0.0713	0.0714	0.0709	0.0600	0.0519
	ESR	0.1507	0.1022	0.1082	0.0952	0.0801
	RCPR	0.1209	0.0810	0.0781	0.0655	0.0539
	Our method	0.0668	0.0642	0.0702	0.0567	0.0497

**(a):** 

	Method	Contour	Eyebrow	Mouth	Nose	Eye
LFPW (68 points)	SDM	0.0718	0.0581	0.0433	0.0363	0.0337
	ESR	0.0746	0.0634	0.0535	0.0435	0.0408
	RCPR	0.0830	0.0615	0.0484	0.0414	0.0367
	Our method	0.0634	0.0518	0.0406	0.0364	0.0332

**(b):** 

	Method	Jaw	Eyebrow	Mouth	Nose	Eye
LFPW (29 points)	SDM	0.0385	0.0381	0.0376	0.0389	0.0295
	ESR	0.0498	0.0419	0.0461	0.0435	0.0350
	RCPR	0.0637	0.0460	0.0471	0.0433	0.0309
	Our method	0.0336	0.0360	0.0365	0.0362	0.0292

**(c):** 

	Method	Jaw	Eyebrow	Mouth	Nose	Eye
COFW (29 points)	SDM	0.0607	0.0643	0.0614	0.0525	0.0457
	ESR	0.1255	0.0873	0.0882	0.0781	0.0664
	RCPR	0.1055	0.0679	0.0633	0.0533	0.0440
	Our method	0.0561	0.0569	0.0603	0.0497	0.0435
